# HIV-associated photodermatitis in African populations

**DOI:** 10.3389/falgy.2023.1159387

**Published:** 2023-05-04

**Authors:** Thuraya Isaacs, Rannakoe Lehloenya

**Affiliations:** Division of Dermatology, Department of Medicine, University of Cape Town, Cape Town, South Africa

**Keywords:** photodermatitis, HIV, photosensitivity, actinic dermatitis, photodermatoses, pigmented skin

## Abstract

Photosensitive dermatoses are seen in 5% of HIV-infected persons. These include drug- and chemical-induced photoallergic and phototoxic reactions, chronic actinic dermatitis of HIV, photo lichenoid drug eruptions, and porphyria. Data on photodermatitis in HIV are limited to case reports and series. The pathogenesis is not completely understood and includes a th2 phenotype in HIV which results in impaired barrier function and resultant allergen sensitisation as well as immune dysregulation. The objective of this manuscript is to review the literature on the clinical phenotype, pathogenesis, role of photo and patch testing, outcomes, and treatment of photodermatitis in HIV in an African population.

## Introduction

Photosensitivity is defined as a pathologic response to non-ionising radiation after normal exposures. On the other hand, photodermatoses refers to a varied group of skin disorders that are caused or exacerbated by an abnormal or excessive reaction to sunlight (1). A good understanding of the pathogenesis of photodermatoses is important as it not only helps establish the diagnosis but guides the management and prevention of complications. Ultraviolet (UV) radiation is the major driver of photosensitivity reactions, although some reactions are seen in response to visible light (2).

## Classification of photodermatoses

Based on the current understanding of pathogenesis, there are varying classifications used for photodermatoses. Some authors include four types, these being immunologically mediated; drug- and chemical-induced, photoaggravated, and photosensitivity associated with defective DNA repair mechanisms (1). Others group them into five types, namely primary; exogenous; photo-exacerbated; metabolic, and genetic photodermatoses (3). There is considerable overlap between these classifications as well as between the individual types themselves as highlighted below.

**i) Immunologically mediated photodermatoses** (IMP) are sometimes referred to as **idiopathic or primary photodermatoses**. However, as the pathogenesis of these disorders is understood, the term idiopathic continues to lose relevance. The main pathomechanism of IMP is thought to be UV-driven autoimmunity. Exposure of normal endogenous components of the body to ionizing radiation alters them structurally to become antigenic and trigger an immune response. Genetic predisposition and environmental factors, beyond exposure to ionizing radiation, are thought to play a significant role. Photodermatoses that are thought to be immunologically mediated include polymorphic light eruption (PMLE), actinic prurigo, hydroa vacciniforme, chronic actinic dermatitis (CAD), and solar urticaria (1, 3).
**ii)** In **drug- and-chemical induced photodermatoses**, the causative chemical is known and is usually exogenous. The resultant reactions are either immunologically mediated (photoallergic) or non-immunologic (phototoxic). Some of the photosensitizing compounds have found a therapeutic role in the management of skin disorders. Examples include psoralen which is combined with UVA to treat a number of skin conditions and methyl aminolevulic acid (ALA) which is used in photodynamic therapy to treat cancerous and precancerous skin conditions (4). Exposure to sunlight is not essential for the development of
**iii) Photoaggravated dermatoses**. However, sun exposure can trigger and/or exacerbate these conditions. Photoaggravated dermatoses include lupus erythematosus, dermatomyositis, Darier's disease, rosacea, and melasma (5–9).
**iv) Genetic photodermatoses** include many due to defective DNA repair mechanisms and metabolic abnormalities. The classic example of defective repair mechanisms is xeroderma pigmentosum, an autosomally recessive inherited disease associated with photosensitivity, premature ageing, and early development of skin cancers. Included in this group are Bloom, Rothmund-Thomson, and Cockayne syndromes. Porphyrias, also genetic photodermatoses, are the best-known
**v) Metabolic photodermatoses**. In porphyria, there is an accumulation to phototoxic porphyrins in the skin amongst other organs. This is due to genetic defects in various enzymes resulting in the accumulation of phototoxic precursors. Porphyria cutanea tarda on the other hand is an acquired form of the disease (10). Porphyrias may be activated by exposure to certain medications or toxins. Hartnup disease also referred to as “pellagra-like dermatosis” is an autosomal recessive metabolic disorder associated with the malabsorption of nonpolar amino acids including tryptophan. Tryptophan is a precursor to nicotinamide, the deficiency of which also manifests as nutritional pellagra, which can occur independent of the genetic defect (11).

## Reaction patterns in photodermatoses

Classically photodermatitis is characterised by the involvement of sun-exposed areas, such as the face, ears, scalp, posterior neck, upper back, “V” area of the chest, extensor arms, and dorsum of hands. It spares the nasolabial fold, supraorbital fold, post auricular and submental area (2). However, there are subtle differences between the different types depending largely on pathogenesis.

**i) Phototoxic reactions** result from direct tissue or cellular injury. It may occur in any individual exposed to a high enough dosage or the wavelength of radiation and does not require previous sensitisation (12). Clinically, phototoxicity presents as an exaggerated sunburn reaction with burning and stinging in the sun-exposed sites within minutes or hours of exposure. Histologically it is characterised by apoptic keratinocytes (sunburn cells) with dermal lymphocytic and neutrophilic infiltrate (13).
**ii) Photoallergic reactions** are less common than phototoxic reactions and usually require a minimal exposure to the photosensitizing drug and prior sensitization (14). A photoproduct acts as a hapten or as a complete antigen to generate a type-IV hypersensitivity reaction. The reaction develops 24 h or more after the initial exposure and is clinically eczematous. If extensive it may extend to include non-sun-exposed skin. Prior contact may not be necessary if the patient has been sensitised to a similar molecule (12, 14). Photoallergic reactions are similar to ordinary allergic contact reactions on histology, the main features being spongiosis and a dermal lymphohistiocytic infiltrate (15).
**iii) Lichen planus and lichenoid reactions** as a whole are a poorly characterised group as is the photolichenoid subset. The unifying feature of these “lichen planus-like” reactions and lichen planus is the presence of an inflammatory pattern histologically characterized by damage to the basal keratinocytes, usually by lymphocytes (16).

## HIV and photosensitivity

Data on photodermatitis in HIV are limited to case reports and small case series and on photodermatitis in HIV-infected Africans it is even more limited. HIV infection is associated with higher odds of developing photosensitivity, and photosensitivity can be a presenting feature of HIV (2, 17, 18). The spectrum of photodermatoses that have been reported in HIV includes PMLE, CAD, photodistributed drug eruptions, photoaggravated granuloma annulare, pellagra, porphyria cutanea tarda, and actinic prurigo (19–24). Actinic lichenoid leukomelanoderma of HIV, a photosensitive eruption observed in South African patients, in the authors' opinion most likely represents CAD in HIV-infected persons (25).

CD4+ T-cell depletion is the hallmark of HIV infection systemically and on the skin. This is due to the active destruction of CD4+ cells by the virus, increased percentage of CD4+ T-cells undergoing apoptosis, reduction in the proliferative capacity of CD4+ T-cells, and an increase in the expression of CD4+ T-cell inhibitory molecules like CTLA-4 and PD-1. The reduction in CD4+ T-cells drives a switch from Th1 to Th2 cytokine polarization resulting in a progressive decline of IFN-γ and cytotoxic T-lymphocyte functioning and an incline in IL-4, IL-5, and IgE (26). The Th2 polarization results in impaired barrier function and resultant predisposition to allergen sensitization. Th17 cells that contribute to epithelial barrier integrity are also targets for HIV infection (27, 28). CD4 expressing cells affected by HIV infection include T regulatory cells (T_reg_) and Langerhan cells. T_regs_ serve an important role as guardians of immunological self-tolerance and prevention of autoimmune diseases. Langerhan cells are the major cutaneous antigen-presenting cells and lead off immune responses during initial antigen exposure by activating resident immune cells as well as linking innate and adaptive immune systems. In HIV infection, there is a compensatory expansion of CD8+ T-cells, terminal effector T cells in the skin in an effort to control ongoing retroviral infection and mediate tissue damage. The overall effect of this immune dysregulation sometimes has a consequence of inducing new skin disorders, including photodermatoses (26, 29).

Persistent T-cell activation is another hallmark of HIV infection. This has been attributed to the persistence of HIV viral reservoirs, even after successful virological control; intestinal microbial translocation into circulation as a result of impaired gut barrier; depletion and functional impairment of Treg cells; and coinfection with or reactivation of other viruses (30, 31). It has recently been established that human T cells are independently photosensitive. Under the influence of light, T cells were triggered to produce hydrogen peroxide (H_2_O_2_), a major source of reactive oxygen species and effector of oxidative stress. Blue light was also found to potently enhance T cell motility, stimulating random cell movement and chemotaxis. Significantly, photosensitivity was greater in activated compared to naïve T cells (32).

Some of the medications used to treat HIV, HIV-associated opportunistic infections, and complications such as trimethoprim-sulfamethoxazole, non-steroidal anti-inflammatories (NSAIDs), and antiretrovirals (ART) can be photosensitizing and increase the risk of photodermatitis. A dose-response association with increasing UV exposure and photosensitivity in HIV-infected individuals has been reported. This suggests that there is variation in the intensity and duration of exposure to initiate and maintain different clinical phenotypes of photodermatitis (2, 17). However, multiple studies have failed to find an association or a change in MED in HIV-infected populations. (17, 23, 33)

## Considerations in pigmented skin

There is an increasing awareness of the underrepresentation of pigmented skin in both undergraduate and postgraduate teaching, textbooks, journal articles, and search engines (34). Photodermatoses are no exception and there is a need to correct this anomaly. Photosensitivity is up to seven times more common in HIV-infected individuals with pigmented skin (2). Globally, Africa carries the heaviest burden of HIV, thus the largest proportion of HIV-infected persons are melanin rich (35). There are numerous factors that may potentially impact the clinical presentation of photodermatoses as well as severity in African populations with higher Fitzpatrick skin types (34, 36). These include (i) detection of erythema and purpura, both being more difficult in melanin-rich skin, (ii) susceptibility to dyspigmentation based on the chromatic tendency theory which seems to be genetically pre-determined and inherited in an autosomal dominant pattern, (iii) contrast between normal skin and dyspigmented skin which has a more detrimental cosmetic outlook for darker skin, (iv) the generally higher preponderance of darker skin to pruritus, a major feature in many photodermatoses (v) pigmented skin has larger mast cell granules and increased activity in some disorders like keloids. Mast cells play a significant role in HIV by promoting infection of CD4+ as well as being associated with increased antigenic responses in HIV-infected patients (34, 37–40).

Diltiazem, a calcium channel blocker is associated with photodistributed hyperpigmentation that seems to be confined to darker skin tones. The exact pathomechansim is unclear but seems to be due to impaired melanogenesis and aberrant transfer of immature melanosomes from melanocytes to keratinocytes (41, 42).

## Clinical spectrum of HIV-associated photodermatoses

Table 1 summarises the clinical and histological features of photodermatoses relevant to HIV-infected persons.

**Table 1:** Summarises the clinical and histological features of photodermatoses relevant to HIV-infected persons.

Type	Clinical	Histology	Remarks
**Immunologically mediated**			
Chronic actinic dermatitis	Eczematous; infiltrated patches and plaques; lichenification; post inflammatory hyperpigmentation and depigmentation	Spongiosis; variable acanthosis; dermal and perivascular lymphocyte; sometimes histiocytes, eosinophils and plasma cell; rarely atypical lymphocytes with epidermotropism	
Actinic lichenoid leukomelanoderma	Circumscribed violaceous, scaly plaques; progressive depigmentation with peripheral hyperpigmentation	Lichenoid band of lymphocytes; irregular acanthosis; saw toothing; apoptotic keratinocytes; pigment incontinence	
Polymorphous light eruption	Intense itch; variable lesions but monomorphic for an individual; erythematous or skin-colored papules or plaques. Sometimes oedematous plaques and blisters	Dermal oedema; perivascular and periadnexal lymphocytes; neutrophils in early lesions; spongiosis and vesicles	Onset spring or early summer or use of tanning bed; recurs annually
**Drug induced**			
Photoallergic	Eczematous; itchy; develops >24 h even on re-exposure	Spongiosis; dermal lymphohistiocytic infiltrate	MED reduced; positive photopatch test
Phototoxic	Exaggerated sunburn; burning and stinging sensations; within minutes or hours of exposure	Apoptotic keratinocytes; dermal lymphocytic and/or neutrophilic infiltrate	MED reduced; negative photopatch test
Photolichenoid	Purple macules, papules; confluent to form patches, plaques; resolve with persistent hyperpigmentation	Apoptotic keratinocytes upper epidermis; parakeratosis; wedge shaped hypergranulosis; saw toothing; lichenoid band of lymphocytes; vacuolar degeneration; pigment incontinence; eosinophils	Normal MED; eosinophils and parakeratosis distinguish it from lichen planus
**Photoaggravated**			
Pellagra	Erythema; evolve to hyperpigmentation; scale; dry; lichenification; fissuring if severe	Pale epidermis; keratinocyte ballooning; epidermal necrosis if severe; little to no inflammatory cells; dilated blood vessels	Association with diarrhoea and delirium
Lupus erythematosus	Circumscribed plaques; erythema; can be annular; scaly. Discoid lupus - follicular plugging; scarring; peripheral hyperpigmentation with central depigmentation	Interface dermatitis; vacuolar degeneration; superficial and deep perivascular and periadnexal lymphocytes; dermal mucin	Positive ANA and specific autoantibodies
**Metabolic**			
Porphyria	Skin fragility; blisters; scarring; milia; hyperpigmentation; hypertrichosis	Cell poor subepidermal blister; festooning of dermal papillae; thickened and hyalinized blood vessel walls	Elevated porphyrin levels in serum, urine, and stool
**Genetic** **#**			

# Too many and too variable.

ANA, antinuclear antibody; MED, minimal erythema dose.

### Chronic actinic dermatitis

Chronic actinic dermatitis (CAD) is a rare persistent and disfiguring photodermatosis, encompassing a spectrum of disorders including actinic reticuloid, photosensitive eczema, and persistent light reactivity. It represents 4.8 to 17% of photodermatoses seen in photobiology clinics (43). Individuals of all skin types can be affected, but it has been reported more commonly in individuals with Fitzpatrick V and VI. There is no data supporting familial inheritance. CAD has a predilection for men over the age of 60 who spend time outdoors, and a history of allergic or photoallergic contact dermatitis to multiple allergens. CAD has also been reported in younger patients with atopic dermatitis. The disease also has a strong association with contact or photoallergy to *Compositae*, a group of plant-based aero allergens (44, 45).

The pathogenesis of CAD is poorly understood. It is postulated to be a delayed-type hypersensitivity reaction to an altered endogenous protein that has been made antigenic by photoinduced reaction (46). Its clinical and histopathological characteristics, and predominance of CD8+ T cells, resembles allergic contact dermatitis and hence a similar pathogenesis can be inferred. The antigenic molecules in CAD have been postulated to be DNA, RNA, or molecules related to these (46, 47). Thus, CAD may be a delayed hypersensitivity response to UV-damaged nucleic acids, triggered by various forms of immunosuppression including ultraviolet (UV) radiation and HIV. Contact dermatitis-induced enhanced immunoreactivity has also been postulated to play a role (46).

The diagnostic criteria of CAD as proposed by Hawk and Magnus are:

• chronic photodermatitis, characterized by a persistent eczematous eruption of infiltrated papules and plaques, in predominantly sun-exposed sites, in the absence of continued exposure to photosensitizers.
• abnormal photo test responses namely reduced minimal erythema dose (MED) to UVA, UVB and/or visible light.
• and histological changes consistent with photodermatosis, with or without lymphoma-like changes (48).

**Figure 1:**
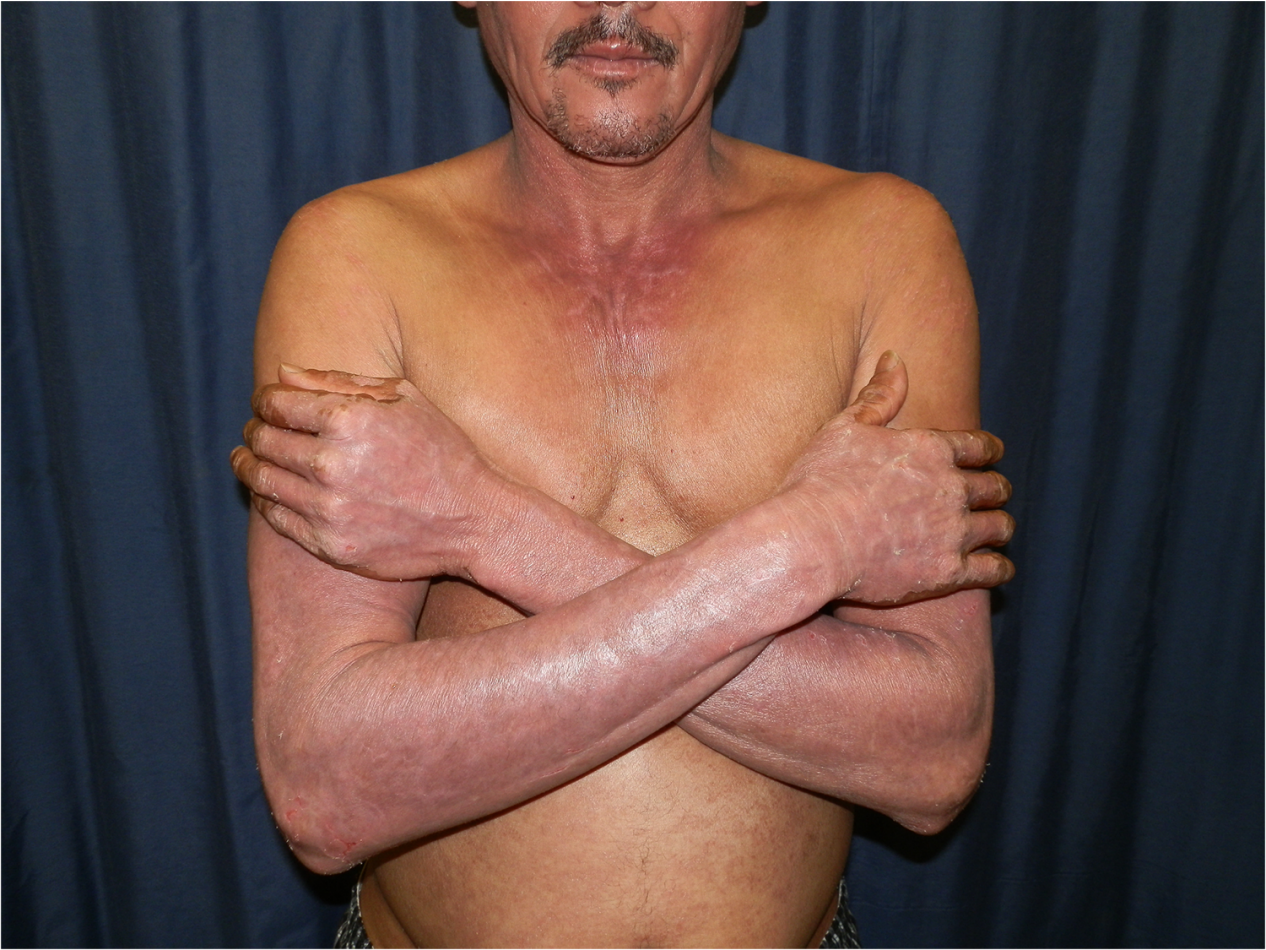
Chronic actinic dermatitis of HIV in Fitzpatrick skin type IV showing a photodistributed eczematous eruption.

Clinically CAD is characterized by pruritic eczematous or lichenified or even infiltrated patches and plaques that are initially limited to sun-exposed areas. Figure 1 Sparing of the nasolabial folds; post auricular, submental, and periorbital areas; skin folds; and interdigital areas is typical (43, 48, 49). In severe cases, CAD may involve sun-protected areas and rarely it can become erythrodermic (50). CAD in darker skin types (Fitzpatrick IV-VI) sometimes results in marked hyperpigmentation that is followed by vitiligo-like depigmentation (49). Other presentations include palmar-plantar hyperkeratosis as well as loss of eyebrows and scalp hair, presumably due to scratching, and (46, 48). Lymphadenopathy and peripheral atypical lymphocytes may be detected in a limited number of patients, reminiscent of Sezary syndrome and cutaneous T-cell lymphoma, creating diagnostic confusion (51–55). On histology CAD is characterized by spongiosis, variable acanthosis with dermal and perivascular lymphocytes (56). There may be atypical lymphocytes with large, hyperchromatic, and convoluted nuclei and epidermotropism. In severe cases, the histological picture may not be distinguishable from cutaneous T cell lymphoma. Histiocytes, eosinophils, and plasma cells may also be present (44, 54, 55, 57). Immunohistochemical studies of CAD biopsies show that most of the skin-infiltrating cells are CD8+ T lymphocytes, with less than 10% showing CD4+ dominant T cell infiltrate in the dermis (55, 56, 58).

**Figure 2:**
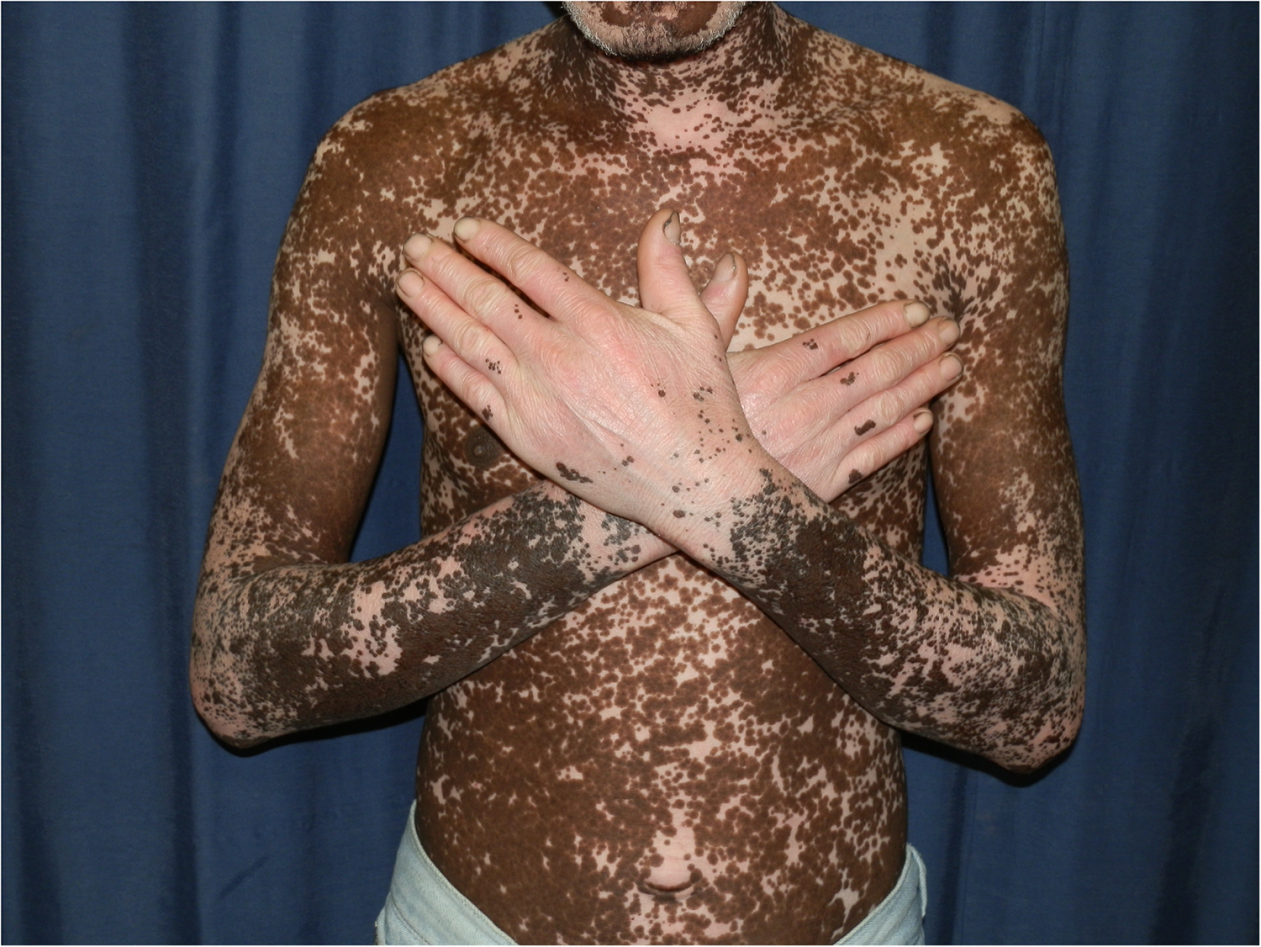
Chronic actinic dermatitis of HIV in Fitzpatrick skin type V with extension to non-sun exposed areas demonstrating hyperpigmentation and extensive depigmentation.

Chronic actinic dermatitis with eczematous features has been described as the presenting illness in patients with HIV infection (18, 19). The clinical features, including distribution and morphology are indistinguishable from CAD in HIV-uninfected persons. In severe cases, HIV-associated late-stage CAD may present with hypopigmented or vitiligo-like depigmentation (19,20). Figure 2 CAD in the setting of HIV seems to predominantly affect men of Fitzpatrick skin types V–VI, which is consistent with observed studies of CAD in HIV-uninfected patients. HIV-associated CAD has also been described in Fitzpatrick skin types III and IV, although less commonly. However, CAD cases in HIV tend to be younger than those who are HIV-uninfected individuals (<50 years vs. >60 years), and usually have significant immunosuppression at presentation (CD4 counts of <200 cells/mm (19, 22). This suggests that HIV, especially advanced disease, hastens the onset of CAD in predisposed individuals and plays a role in its pathogenesis (22).

Like CAD in general, the pathogenesis of HIV-associated CAD has not been defined. CD8+ T lymphocytes are thought to play a central role. A decrease in CD4:CD8 ratio in lesional skin has been reported in all forms of CAD (18, 46, 55, 56). On treating the skin, with associated clinical improvement, there is a reversal of CD4:CD8 ratio in both the skin and blood (18, 56). Pappert et. al. speculated that the decreased CD4:CD8 ratio in HIV results in immune dysregulation and loss of control of anti-self-responses and proliferation of photo-induced self-antigens. The shift towards CD8+ T cell phenotype enhances cytotoxic activity against these self-antigens (18). However, a recent study has shown that these infiltrating CD8+ T cells rarely express cytotoxic molecules like TIA-1, granzyme B, or granulysin (56). The implications then of the diminished cytotoxic markers in CD8+ lesional skin on this hypothesis is not clear. Additionally, there is evidence to suggest that in CAD there may be an acquired quantitative or qualitative defect in Tregs resulting in unchecked cytotoxic T cell responses (59).

### Actinic lichenoid leukomelanoderma of HIV

Actinic lichenoid leukomelanoderma of HIV is a photosensitive eruption anecdotally observed in the South African clinical setting (25). The condition presents in the setting of advanced HIV with low CD4 counts and absence of other photosensitising medication. Clinically it is characterised by well-circumscribed, photodistributed scaly plaques resembling discoid lupus erythematosus. The lesions characteristically start as violaceous macules, which become progressively depigmented with peripheral hyperpigmentation, sometimes with scale (25). While it remains to be established whether this condition is a distinct entity, it is also possible that this condition falls along the spectrum of HIV photodermatitis with vitiligo-like depigmentation or CAD in HIV-infected persons (60, 61). Further studies are needed to characterise this condition.

### HIV photodermatitis with depigmentation

HIV photodermatitis with widespread vitiligo-like depigmentation is rarely reported (60, 61). We reported a photodistributed lichenoid drug eruption with depigmentation in an HIV-infected man on treatment for a second episode of tuberculosis. The rash initially developed during the first course of tuberculosis treatment and resulted in areas of depigmentation and hyperpigmentation. On reexposure to the same regimen to treat the second episode of tuberculosis, there was recurrence with violaceous patches within the depigmented areas from the first episode. On completion of the second regimen, repigmentation was considerably better than after the first episode. Phillips et al. reported a 60-year-old man ART naïve man with advanced HIV (CD4 count of 7 cells/µl) who presented with a photodistributed, pruritic eruption associated with extensive depigmentation surrounded by hyperpigmentation. Histology revealed a spongiotic dermatitis with abundant eosinophils. A similar case was described involving a Ugandan woman with advanced HIV (CD4 count of 2 cells/µl) occurring one month after initiation of ART with stavudine, lamivudine, and nevirapine (60, 61). Most areas repigmented after the regimen was changed to zidovudine, lamivudine, and efavirenz. The authors postulated that the photodistributed depigmentation was due to a photosensitizing effect of either the ART or cotrimoxazole; with the ART being more likely. Histology was not reported (60). All three cases were of Fitzpatrick skin phototype V or greater (62).

Depigmentation has also been reported in chronic lichenoid photo eruptions in HIV patients with a low CD4 T-lymphocyte count <50 cells/µl (63, 64). We reported an HIV-infected man with low CD4 count and multidrug-resistant tuberculosis developed a generalised lichenoid eruption that progressively worsened and depigmented after initiating tuberculosis treatment. Treatment was continued under the cover of sun protection, potent topical corticosteroids, and PUVA to induce hardening. He completed nine months of treatment and soon afterwards the depigmentation reversed gradually (65).

Photosensitivity with depigmentation prior to the diagnosis of HIV, has also been reported. The presentation was marked by scaling, erythema, and concurrent vitiligo-like depigmentation of the face, arms, and hands. The condition improved with the use of sunscreen and topical steroids (2).

The differential diagnosis of photodermatitis with vitiligo-like depigmentation includes discoid lupus erythematosus (DLE), vitiligo, and lichen simplex chronicus. To differentiate from DLE there is an absence of scarring and histology does not demonstrate an interface dermatitis. The immunodeficiency, photo distribution, and histology demonstrating spongiotic dermatitis with eosinophils distinguish HIV photodermatitis from vitiligo. Likewise, the photo distribution and absence of lichenification exclude lichen simplex chronicus.

### Actinic lichen planus

Actinic lichen planus is a morphologic variant of LP that predominantly affects sun-exposed areas and has a high prevalence in darker skin. Three variants of actinic LP have been described, namely annular, pigmented, and dyschromic, in that order of their frequency. The annular variant presents as annular erythematous brownish patches or plaques with or without atrophy. The major feature of the pigmented variant is the presence of melasma-like pigmentation. Dyschromic-type actinic lichen planus presents as whitish pinhead-sized and coalescent papules (66, 67). HIV seems to have a stronger association with photolichenoid eruptions. In a series of 32 patients with histologically confirmed lichenoid eruption or photodermatitis, 12/32 were HIV infected, and in all 12, the lesions were photodistributed (63).

### Drug induced photodermatoses

Photosensitive drugs are chromophores that absorb photons and undergo chemical reactions. The chemical structure of the chromophore determines the wavelengths of radiation it absorbs, with most reactions being caused by UVA rather than UVB. Drug-induced photodermatoses in the setting of HIV include phototoxic, photoallergic, and photolichenoid reactions. Drugs most commonly associated with photosensitization include amiodarone; chlorpromazine; thiazide diuretics; tetracyclines; quinolones, particularly nalidixic acid; nonsteroidal anti-inflammatory drugs derived from propionic acid, voriconazole and vemurafenib, a B-Raf enzyme inhibitor used in the management of late-stage melanoma (68).

**Figure 3:**
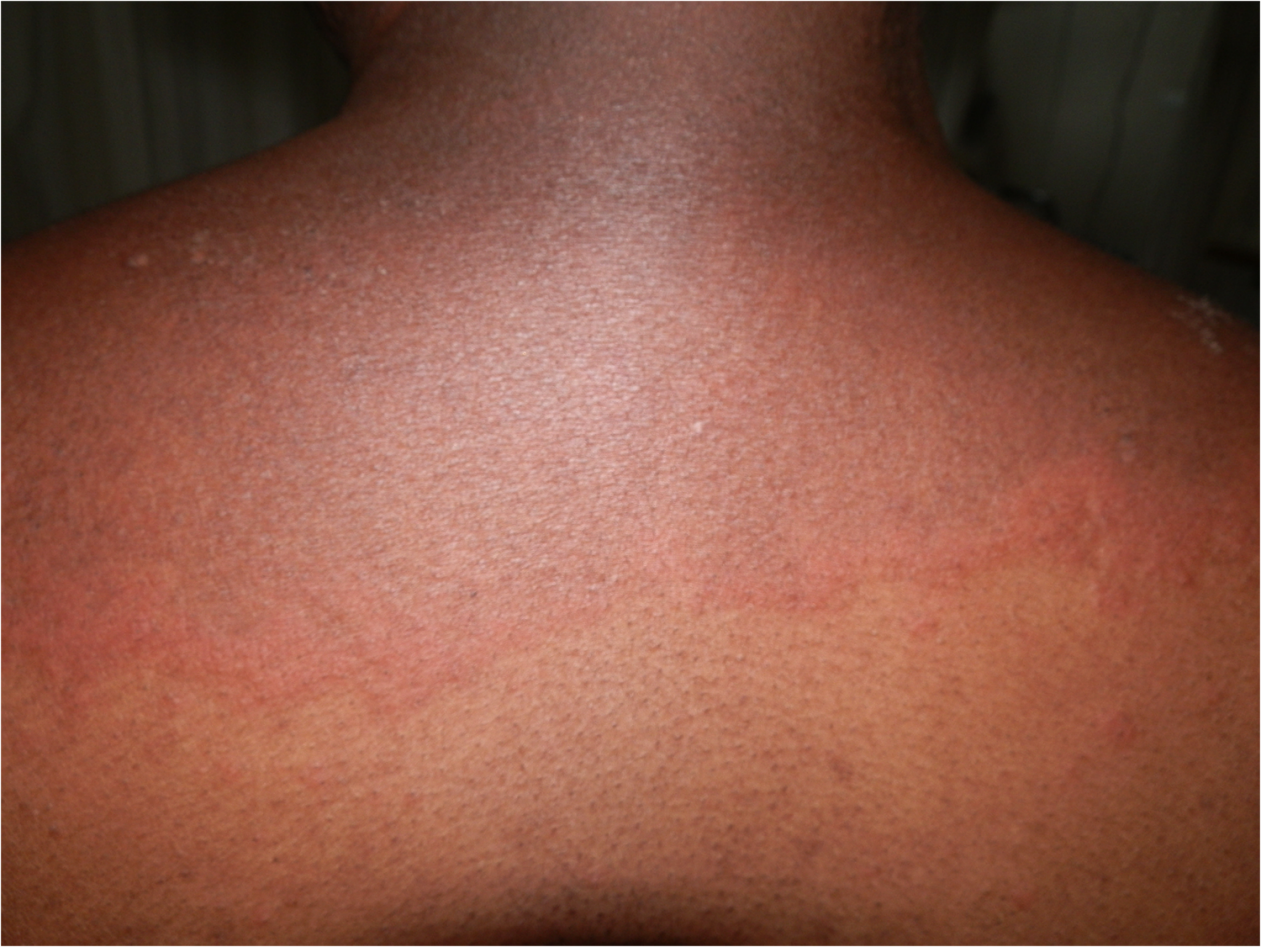
Efavirenz-associated reaction demonstrating in photodistributed indurated erythema with sharp cut off and annularity.

**Figure 4:**
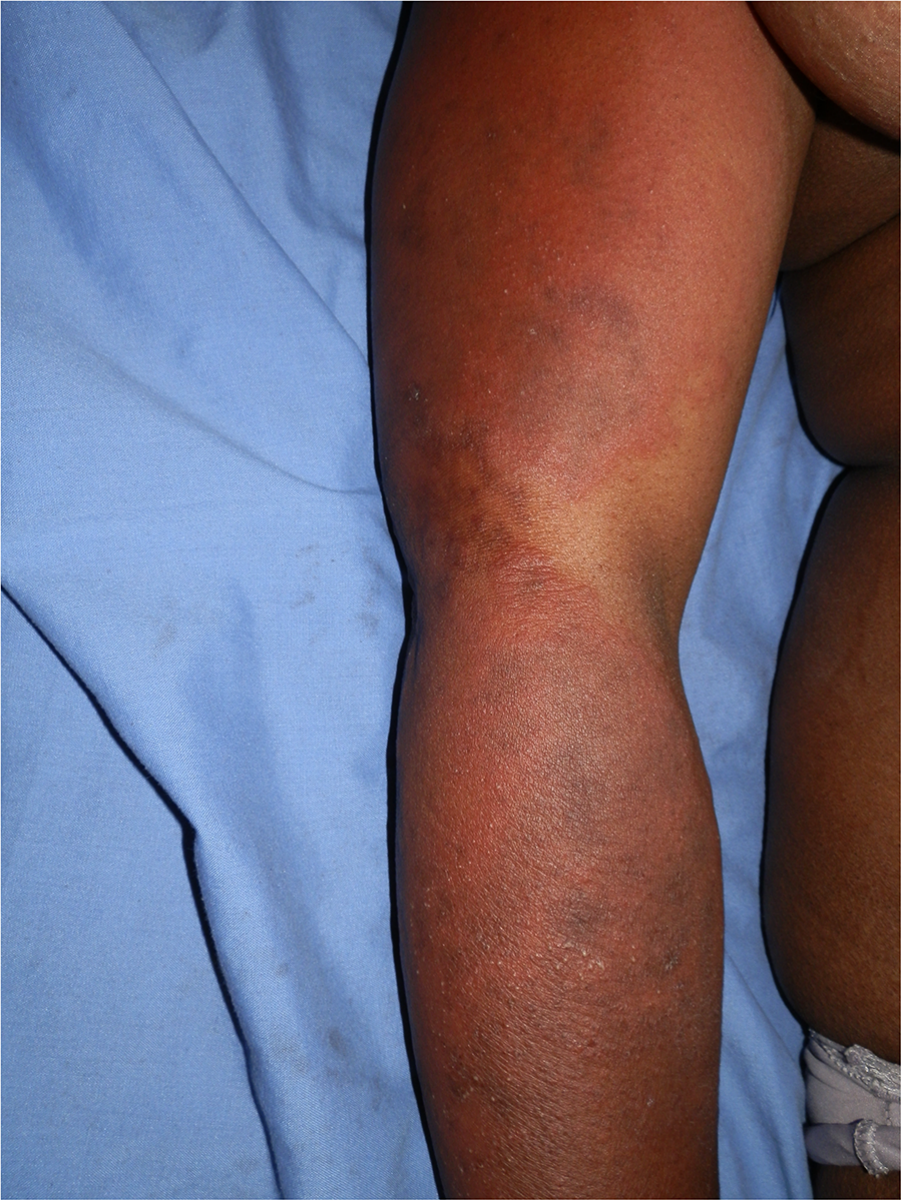
Efavirenz-associated reaction demonstrating in photodistributed indurated erythema with annularity of plaques and dusky centres.

ART, antituberculosis drugs, sulphonamides, and antifungals, all drugs used to treat HIV and associated opportunistic infections, have at least one drug or a class of drugs that has been reported to be a photosensitizer. Efavirenz, an ART drug, has been reported to cause a photodistributed transient eruption (69–71). A small series of five cases in South Africa reported photodistributed annular plaques with an indurated erythematous edge as the most common presentation of the efavirenz reaction. Figures 3, 4 Histology findings were non-specific. Systemic features were mild and included a mild elevation in alanine and/or aspartate transaminases. Despite the continuation of efavirenz, the majority resolved (72, 73). Cotrimoxazole has been implicated *in vitro* as a photosensitizer, and the sulfamethoxazole component is believed to be responsible (74). Dapsone photoallergic dermatitis has been described in case reports (75–77). Despite its use as an alternative to cotrimoxazole for *Pneumocystis jirovecii* prophylaxis, there are no reports of dapsone photoallergic dermatitis in African people or HIV patients.

Antifungals known to cause photosensitivity include voriconazole, itraconazole, ketoconazole, and griseofulvin (78–81). Itraconazole reactions include a sunburn-like reaction, most likely phototoxic with decreased MED and negative photopatch tests (78). Voriconazole photosensitivity includes sunburn-like erythema, hand hyperpigmentation, linear papulo-vesicular lesions, erythroderma, discoid lupus erythematosus, actinic cheilitis and pseudoporphyria (78, 82). Most reactions occurred in patients receiving long-term immunotherapy, however, there is no data reported in the HIV infected population. There are reports of the development of squamous cell carcinoma and melanoma in voriconazole-induced sites of photosensitivity (83). Amongst the drugs used to treat tuberculosis, photodistributed eczematous eruption due to isoniazid and pyrazinamide has been described (84). On discontinuing the drug, the photosensitivity usually resolves. However, persistent photosensitivity and progression to CAD have been reported (12, 14).

Photolichenoid drug reactions seem to be more common in HIV-infected people than in the general population and in this setting often pose major management challenges. In a case series of 32 patients in San Francisco, with a histologic diagnosis of lichenoid eruption or photodermatitis, 12/32 were HIV infected, and all 12 were photodistributed. Exposure to known photosensitizing drugs preceded the eruption in 10/12. Patients with pigmented skin and advanced HIV disease (CD4 < 50 cells/µl), were disproportionately affected. Histologically 9/12 showed lichenoid reaction, 2/12 had features of lichen nitidus, and 1/12 was eczematous. Two of those with lichenoid reactions on histology developed marked depigmentation. No cases of lichen planus were found in the HIV-infected group. The authors suggested that previous cases of classic lichen planus in HIV may represent lichenoid photodermatitis (63).

**Figure 5:**
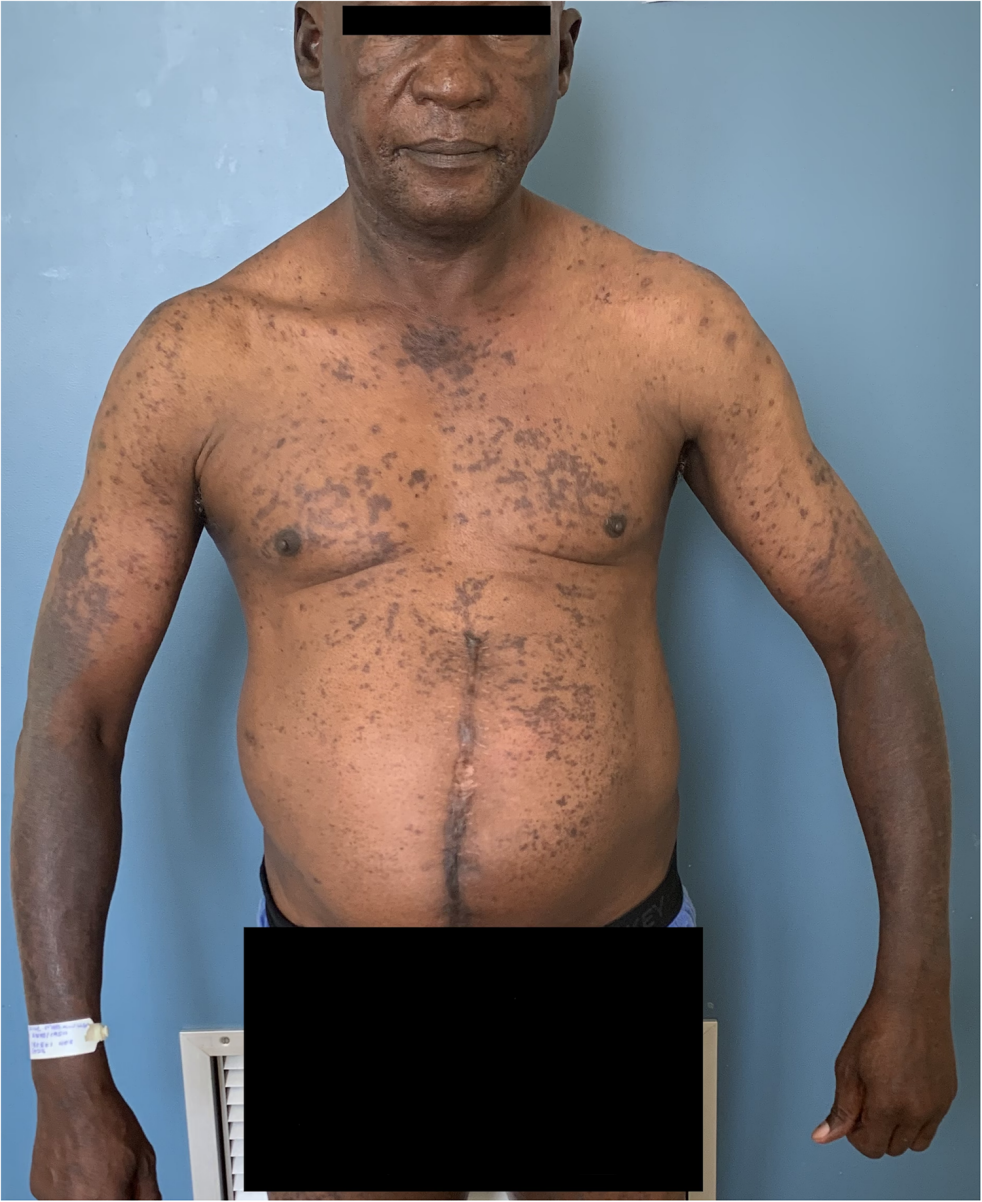
Photolichenoid drug eruption to first line antituberculosis treatment demonstrating violaceous patches, plaques.

**Figure 6:**
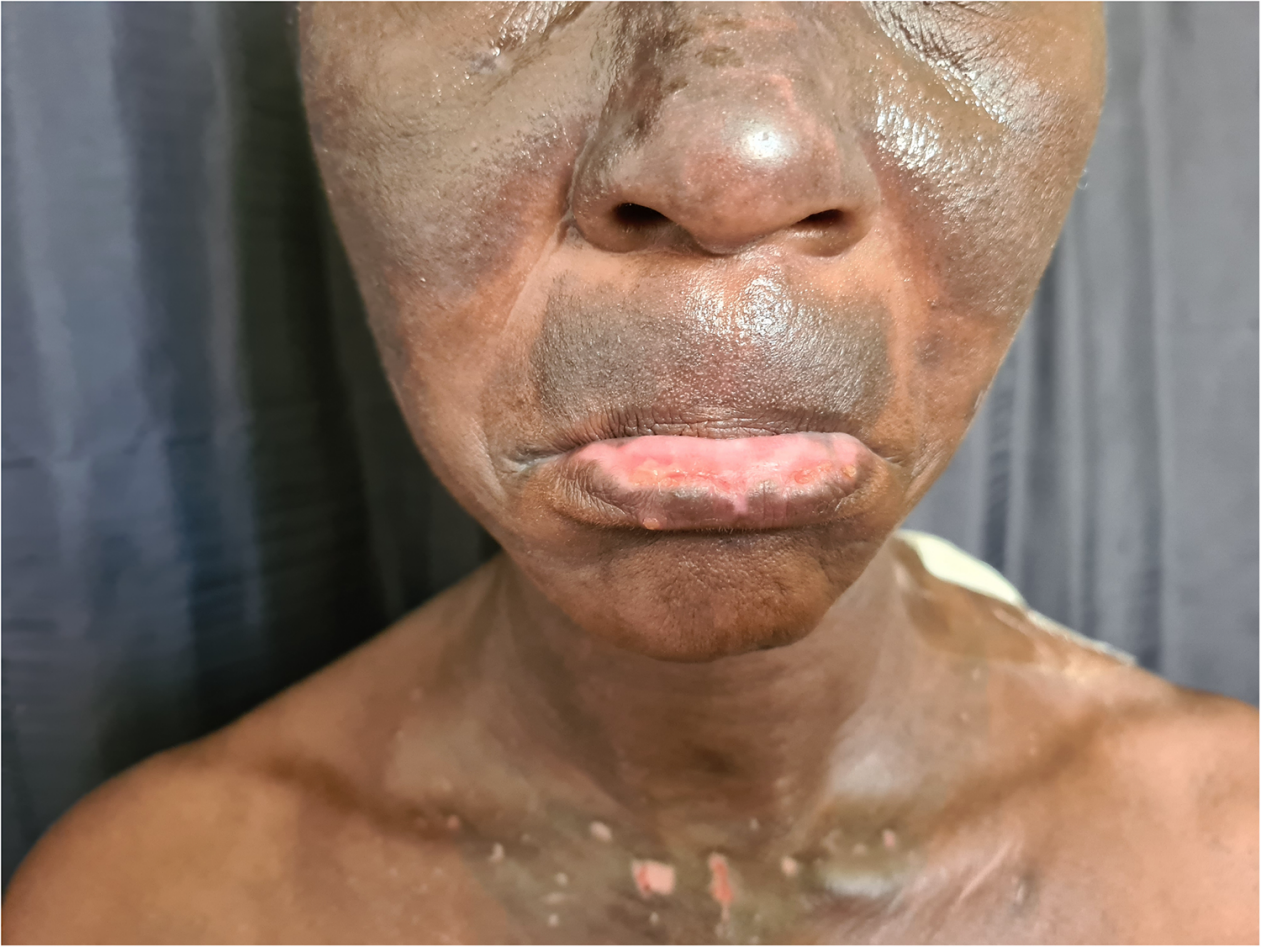
Photolichenoid drug eruption to first line antituberculosis treatment demonstrating violaceous patches and plaques with depigmentation.

Anti-tuberculosis medications, including isoniazid and pyrazinamide, have been reported to cause a lichenoid and/or photo lichenoid eruption (65, 85, 86). The pathogenesis is unclear but may be due to delayed hypersensitivity. LDR presents as purple itchy papules becoming confluent and hyperpigmented with continuing exposure to the offending drug. It is often photodistributed and lacks mucosal involvement Figures 5, 6. The interval between drug initiation and the rash ranges from days to years, with most cases occurring within months. On drug withdrawal, the lesions resolve with persistent hyperpigmentation, often lasting for many years. Although photolichenoid eruptions may be related to photosensitizing agents, there seems to be no association or changes in minimal erythemal dose (17, 23, 33). Photolichenoid reactions have been reported for isoniazid, confirmed by positive photopatch testing and oral rechallenge (86). Due to the lack of acute markers and delayed resolution of symptoms and signs of the reaction it is often difficult to identify the offending drug. Due to the relative urgency of treating tuberculosis in HIV and the limited number of effective safe drugs, all the drugs are sometimes continued with supportive care until completion of treatment in those with active tuberculosis (62, 65).

### Porphyria cutanea tarda

**Figure 7:**
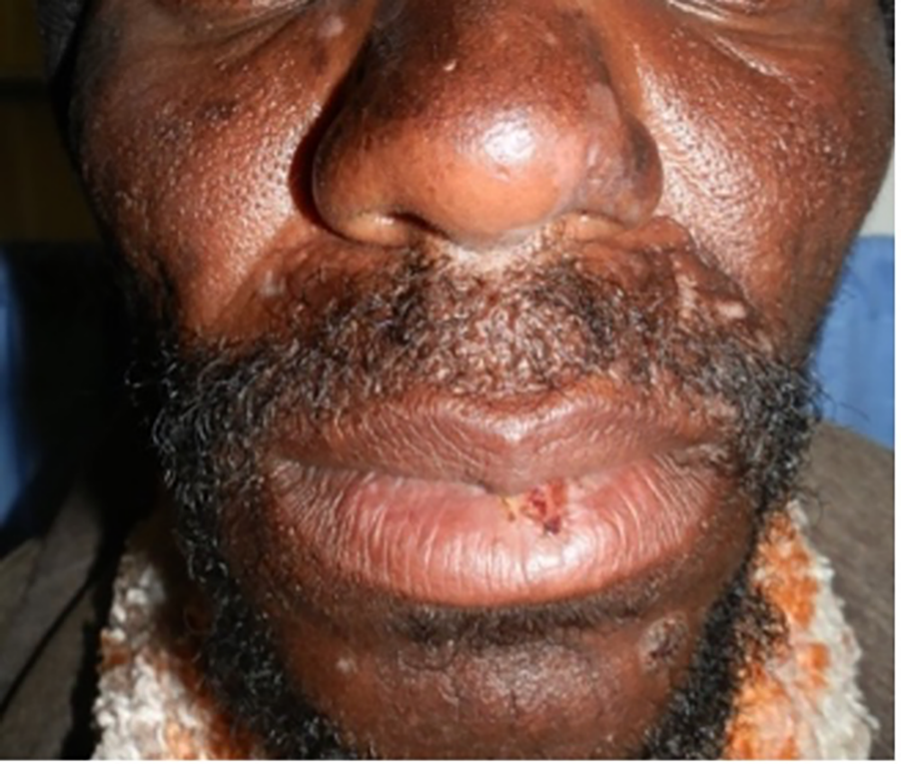
Blisters, erosions, scarring and hyperpigmentation on the face of a patient with porphyria cutanea tarda.

**Figure 8:**
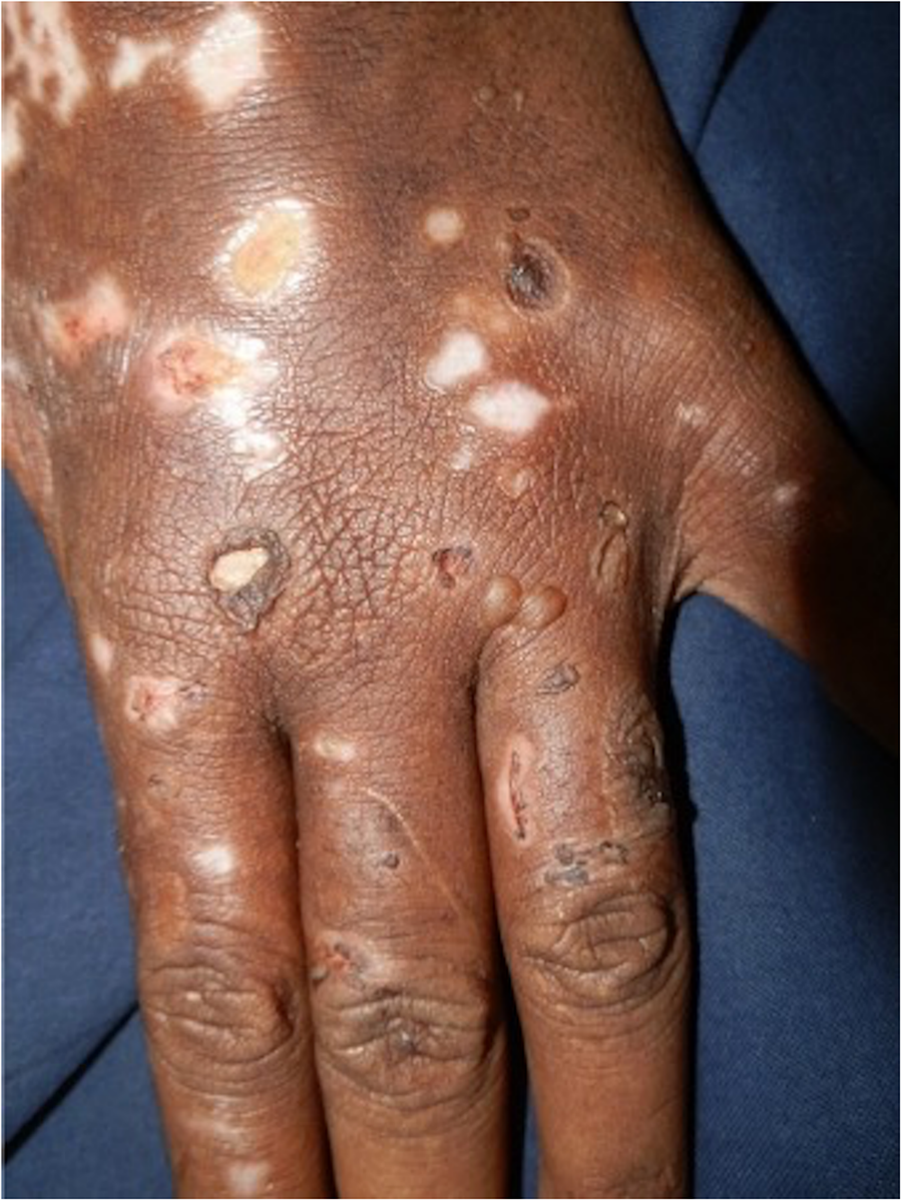
Blisters, erosions, and scarring on the dorsa of the hand of a patient with porphyria cutanea tarda.

Porphyria cutanea tarda (PCT), the most common form of porphyria, is caused by a deficiency of the fifth enzyme of the heme biosynthesis pathway, uroporphyrinogen decarboxylase (UROD). Decreased UROD activity leads to the overproduction of porphyrins in the liver. PCT is a cause of chemical-induced phototoxicity caused by sunlight interacting with endogenous porphyrins within the body. Clinically, these manifest in the third to fourth decade of life with photosensitivity, skin fragility, and blisters. The blisters occur in photodistributed sites particularly the face, V of the neck, and dorsa of the hands, and are associated with scarring and milia Figures 7, 8. Other features include hyperpigmentation, hypertrichosis, and rarely sclerodermoid changes. Earlier studies suggested a causal association between HIV and PCT (87, 88). While the mechanism of PCT in HIV is still not fully understood, it is thought to be due to changes in porphyrin metabolism and liverinjury in the setting of co-infection with hepatitis C. It is now postulated that in most HIV-infected patients with PCT, hepatitis C and not HIV may induce a decrease in UROD activity (89). Drug exposure influences PCT. Amongst first-line antituberculosis drugs, rifampicin is the most likely to be associated with PCT (90, 91). We recently encountered an HIV-infected man who presented with PCT after initiating rifampicin, isoniazid, pyrazinamide, and ethambutol. Based on published reports, we decided to replace rifampicin with rifabutin, a similarly effective rifamycin with less effect on the liver (92). The photosensitivity and blistering improved until he completed the remaining four months of his six-month course of treatment.

Diagnosis of PCT can be confirmed by histology and porphyrin levels. Histologically, PCT is characterized by a cell-poor subepidermal blister, festooning of the dermal papillae, and thickening and hyalinization of the dermal blood vessel walls. Additional features include caterpillar bodies and dermal sclerosis and a mild infiltrate of perivascular mononuclear cells in the upper dermis. These features are not specific to porphyria and can also occur in pseudoporphyria syndromes. The most important diagnostic test to diagnose porphyria remains porphyrin levels in serum, urine, and stool (93).

### Pellagra

**Figure 9:**
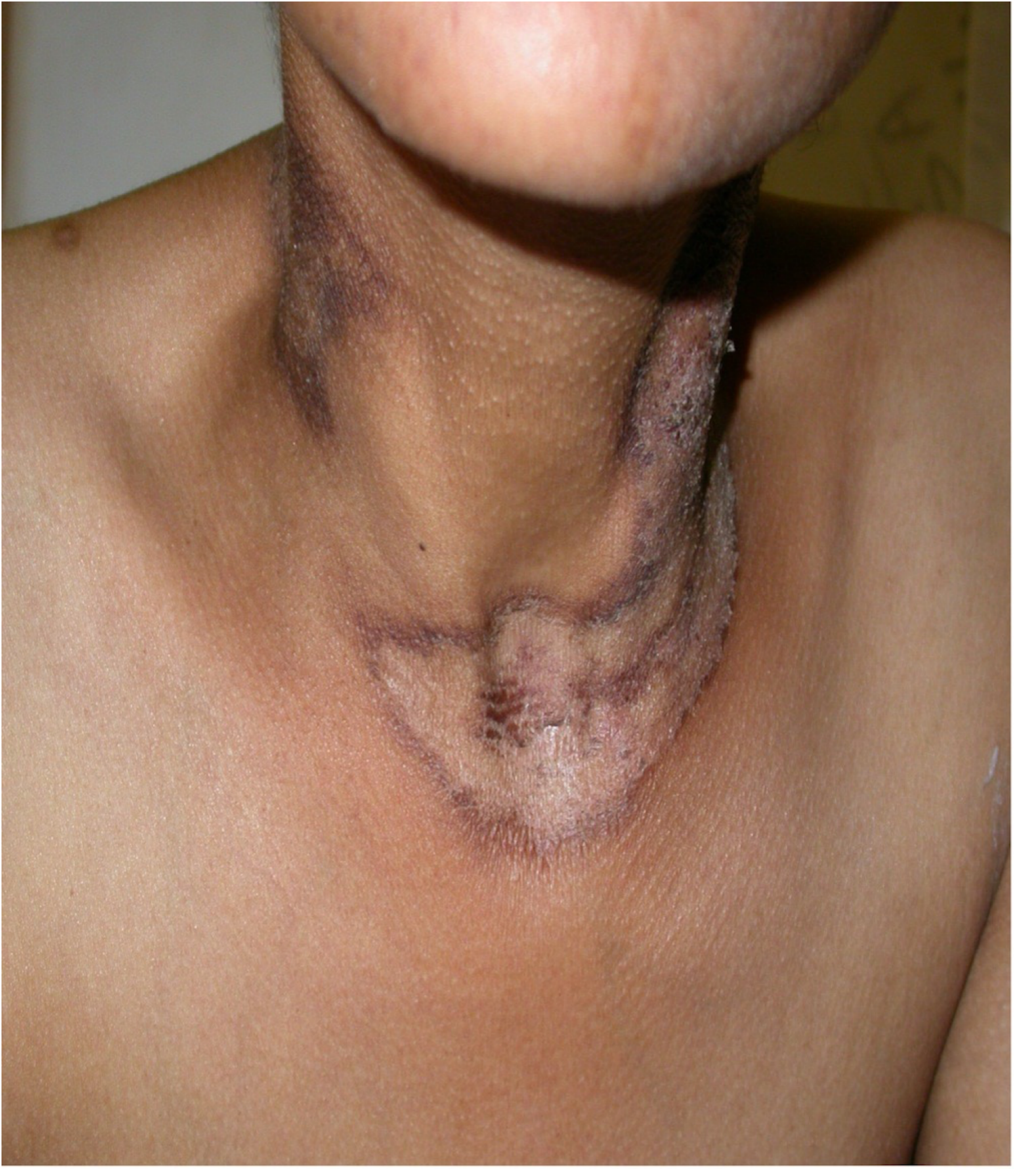
Casal's necklace as a manifestation of pellagra in an HIV-infected woman.

Pellagra, as mentioned earlier is a nutritional disease caused by the deficiency of niacin (also known as vitamin B3). Clinically pellagra is characterized by photodermatitis with gastrointestinal symptoms and neuropsychiatric ailments. Pellagra can be fatal if not timeously recognized and treated. Erythema is the initial clinical feature, and this evolves into hyperpigmentation, dryness, scale, lichenification, and fissuring. On the dorsum of the hands and forearms this is referred to as the gauntlet or glove sign. Asimilar feature on the lower extremities is called the boot sign. The development of fissures on the hands and feet surface is called goose skin. The eruption surrounding the base of the neck is called the Casal's collar or necklace Figure 9. In severe cases blistering occurs within the lesions and this is referred to as wet pellagra which tends to heal with scarring. Additionally, lesions may develop over bony prominences, perineum, and scrotum. The eruption usually presents as erosions with burning pain that intensifies on palpation (94). HIV as well as isoniazid, pyrazinamide, and ethionamide have been shown to cause pellagra or pellagra-like disease (95, 96). It is thus even more important to recognize and prevent pellagra in HIV-infected people on treatment for tuberculosis, particularly breast-feeding women and young children. Breastfed infants receiving isoniazid should also receive pyridoxine 1 mg/kg daily (97).

## Long-term sequelae of HIV-associated photodermatitis

There is a general consensus that chronic photosensitization is associated with an increase in the development skin cancer. This risk is determined by the Fitzpatrick skin type, age at which photosensitizer was used, duration and severity of photosensitization, immunosuppression, the photosensitizing agent/drug, and spectrum of UV absorption amongst others (98). Azathioprine, a photosensitizing immunosuppressant, used after solid organ transplant and inflammatory disorders is associated with an increased risk of squamous cell carcinoma (SCC) by additionally producing mutagenic reactive oxygen species on the skin and an additional risk of SCC on the skin. In transplant patients, the risk is estimated to be higher by the magnitude of 65–250 times (99). This is still applicable when comparing immunosuppressive regimens that incorporate azathioprine and those that do not (100, 101). On the other hand, mycophenolate mofetil, a non-photosensitizing immunosuppressant of similar potency, reduces the incidence of SCC in this setting (99). Other photosensitizing drugs that have been associated with an increased risk of skin cancer include thiazides, angiotensin-converting-enzyme inhibitors, cotrimoxazole, tetracyclines, and azoles (98, 102). On the other hand, long-term use of NSAIDs including those that are photosensitisers, seems to reduce the risk of developing cutaneous SCC (102). Photosensitivity also has a direct relationship with photoaging (103, 104). Concerns about the adverse effects of using light therapies like lasers are not supported by any published reports. This further supports the view that photosensitivity as described is wavelength specific (105).

A recent systematic review that included 19 studies found that between 31% and 39% of photosensitive patients suffered a very large impact on their quality of life. Employment, education, social and leisure activities, and clothing choices were most affected. The study also confirmed that the levels of anxiety and depression were twice those of the general population. Involvement of the face, being female, and an earlier age of onset were associated with significantly more severe psychological morbidity. This study confirmed findings from multiple previous studies and highlights the hidden toll of photosensitivity on sufferers (106, 107). There are limited data on the psychosocial impact of photosensitivity focusing on pigmented skin. The additional impact of more severe dyspigmentation is likely to exacerbate the psychosocial impact of photosensitivity in this population, more so if HIV-infected as both these are independent predictors of poorer quality of life and psychosocial morbidity (108–111).

## Management of photodermatosis in HIV

A good history, clinical examination, a skin biopsy, and photo testing are helpful in identifying photodermatitis and in distinguishing phototoxic from photoallergic reactions. However, this distinction may be difficult as there is sometimes an overlap. Photo testing and photopatch testing may be helpful in assessing patients with photodermatitis and have proven useful to differentiate the photosensitivity mechanism (112). Photo testing, the aim of which is to determine whether the minimal erythema dose (MED) is reduced in the presence of the drug, can be conducted with the use of a solar simulator or in resource-limited settings with the crude method of exposure to midday sunlight. It is done in a number of shielded and unshielded areas on the upper back of the patient while taking and then not taking the suspected drug (13). A reduced MED indicates photosensitivity.

Photo patch testing is an important tool in diagnosing photoallergic drug reactions. It involves the application of drugs on the patient's back and then occlusion. Individual Finn chambers used in standard patch testing may be used. The patches are uncovered after 24 h and irradiated with UVR below the pre-determined MED. The patch is then read 24 h post UV irradiation. A positive photo patch test implies a photoallergic reaction. Although used in the clinical setting, its use for the diagnosis of photo drug reactions due to systemic medication has not yet been validated (13).

Most photosensitivity drug reactions resolve with sun avoidance and drug discontinuation. Patients who are unable to discontinue the offending agent, need multiple photoprotective measures including sun avoidance, protective clothing, broad protection sunscreen, and potent topical steroids. Treatment is often difficult. Phototherapy has been used with success to induce hardening (65). Treatment with thalidomide has been successful in refractory cases (113, 114). However, it is important to note that thalidomide has been reported to increase the HIV viral load, although the clinical significance of this phenomenon is unknown. Therefore, the HIV RNA levels should be monitored after the first and third months of thalidomide treatment and should subsequently be repeated every three months (115).

## Conclusion

HIV photodermatitis is a presenting feature of a myriad of clinical conditions, which should be considered in patients with HIV/AIDS who present with a pruritic, photodistributed eruption. Drug-induced photosensitivity remains a frequent clinical concern. Among the photosensitizers include such drugs as efavirenz, cotrimoxazole, INH, PZA, and antifungal agents. Photo testing may be useful in establishing photosensitivity in both drug-induced and non-drug-induced photodermatitis. Additionally, photo patch testing may be useful to establish the causative agent. Photodermatitis has a profound impact on quality of life. Treatment includes stopping potential photosensitisers, sun avoidance, photoprotective clothing, broad-spectrum sunscreens, and potent topical steroids.
